# Vitamin K Status in Chronic Kidney Disease

**DOI:** 10.3390/nu5114390

**Published:** 2013-11-07

**Authors:** Kristin M. McCabe, Michael A. Adams, Rachel M. Holden

**Affiliations:** 1 Department of Biomedical and Molecular Sciences, Queen’s University, Kingston, ON K7L 3V6, Canada; E-Mails: 5km12@queensu.ca (K.M.M.); adams@queensu.ca (R.M.H.); 2 Department of Medicine, Queen’s University, Kingston, ON K7L 3V6, Canada

**Keywords:** vitamin K, chronic kidney disease, renal insufficiency, vitamin K-dependent proteins

## Abstract

The purpose of this review is to summarize the research to date on vitamin K status in chronic kidney disease (CKD). This review includes a summary of the data available on vitamin K status in patients across the spectrum of CKD as well as the link between vitamin K deficiency in CKD and bone dynamics, including mineralization and demineralization, as well as ectopic mineralization. It also describes two current clinical trials that are underway evaluating vitamin K treatment in CKD patients. These data may inform future clinical practice in this population.

## 1. Introduction

In 2004 it was estimated that over 19 million Americans [1] and over 2 million Canadians [2] had chronic kidney disease (CKD). Compared to the general population, patients with CKD are at marked increased risk for cardiovascular disease and bone fracture. Vitamin K is a composite term referring to a group of fat-soluble vitamins that function as a co-factor for the enzyme γ-glutamyl carboxylase (GGCX), which activates a number of vitamin K-dependent proteins (VKDPs) that are involved in proper hemostasis, as well as vascular, and bone health. We and others have demonstrated that there is a very high prevalence of vitamin K deficiency within the CKD population suggesting that this represents a population at risk for any biological consequences of poor vitamin K status [3,4,5,6].

Vitamin K exists naturally as vitamin K_1_ (phylloquinone) and several forms of vitamin K_2_ (menaquinones) which differ in length of the isoprenoid side chain (MK-4–MK-11) [7]. Vitamin K1 is found in highest concentration in green leafy vegetables, whereas vitamin K_2_ is found in high concentrations in Natto, a popular Japanese food, as well as in some fermented cheeses. Both vitamin K_1_ and MK-4 are found in tissues. Vitamin K_1_ is the major dietary form, whereas MK-4 is believed to be produced via endogenous conversion of K_1_, MK-7 and MK-4 itself. Vitamin K_1_ is present in a variety of tissues with the highest levels being found in the liver [8,9], where it is involved in the carboxylation of coagulation factors (e.g., prothrombin). MK-4 is also found ubiquitously but is found in high concentrations in extra-hepatic tissues including the vasculature and bone. MK-4 appears to be the major form involved in catalyzing the carboxylation of the calcium-binding proteins, matrix Gla protein (MGP) and osteocalcin (OC), which protect against vascular calcification and bone loss, respectively [10]. Accordingly, measurements of inactive or uncarboxylated VKDPs such as uncarboxylated prothrombin, (protein induced by vitamin K absence/antagonism II/PIVKA-II), uncarboxylated osteocalcin (ucOC), and de-phosphorylated uncarboxylated MGP (dp-ucMGP) are used as a functional measure of overall vitamin K status [7,11]. It has been reported that there are high levels of ucOC and dp-ucMGP across the spectrum of low GFR [11]. Therefore, vitamin K may represent a modifiable risk factor for both cardiovascular and bone disease in the CKD population and could represent an important therapeutic target.

## 2. Basic Science

It has been established that vitamin K plays a role in the inhibition of calcification in animal models [12]. In healthy rats, global antagonism of vitamin K with a supra-therapeutic dosage of warfarin led to vascular calcification [12]. Further, the use of dietary vitamin K supplementation prevented and, in some cases, reversed vascular calcification in this model [13,14]. We have recently reported that changing vitamin K status greatly altered vascular calcification in rats with CKD. In contrast to the earlier models utilizing supra-therapeutic warfarin in healthy rats, we demonstrated, in animals with CKD, that low levels of warfarin (generating an INR of between 2 and 3), without any vitamin K supplementation depleted tissue vitamin K concentrations and markedly increased the susceptibility of all vessels to vascular calcification [9]. However, a therapeutic dosage of warfarin did not induce vascular calcification in healthy rats, despite the marked depletion of vitamin K within extrahepatic tissues, suggesting that vitamin K status was critical only when CKD was also present. Further, high dietary vitamin K_1_ significantly increased tissue vitamin K_1_ concentrations and reduced the incidence and severity of vascular calcification in this CKD model [9]. These data indicate that, in the setting of experimental CKD, vitamin K status is an important regulator of vascular calcification [9].

## 3. Clinical Science

### 3.1. Vitamin K Status in Patients with CKD not yet Requiring Dialysis

Insufficient vitamin K availability leads to a decrease in the carboxylation of vitamin K dependent proteins including prothrombin, osteocalcin (OC), and matrix gla protein (MGP) in addition to low levels of circulating phylloquinone (<0.4 nM). Two studies have addressed the vitamin K status of patients with CKD not yet requiring dialysis. We demonstrated in a study of 172 stage 3 to 5 CKD patients, most of whom were well-nourished, that over 50% consumed less than the recommended Adequate Intake (AI) for vitamin K [15]. Further, 6% were vitamin K insufficient according to low levels of serum phylloquinone (<0.4 ng/mL), 60% were insufficient according to high levels of ucOC (>20% ucOC), and 97% were insufficient according to high levels of PIVKA-II (>2 nmol/L) [15]. In this study, lower dietary intake of vitamin K predicted lower levels of phylloquinone and higher levels of PIVKA-II [15]. Schurgers *et al.*, measured dephosphorylated, uncarboxylated MGP (dp-ucMGP) in a cohort of 107 patients ranging from CKD stages 2-5D, and reported that levels of plasma dp-ucMGP increased progressively with CKD stage, indicating a decline in this biomarker with a reduction in GFR [11]. These two studies confirm that patients with early stage CKD, including those who are well-nourished, are at risk for subclinical vitamin K deficiency; the degree to which biomarkers of vitamin K status change in response to supplementation with either vitamin K1 or vitamin K2 in early stage CKD has not been studied.

### 3.2. Vitamin K Status in Patients with CKD Receiving Hemodialysis (HD)

Studies have consistently demonstrated that there is a very high prevalence of sub-clinical vitamin K deficiency in patients requiring HD [3,4,5,6]. Due to its lipophilic characteristics, vitamin K would not be expected to be removed via the dialysis procedure. It is only recently that dietary intake of vitamin K was formally assessed in a cohort of HD patients [6]. In a study of 40 maintenance HD patients, dietary intake of vitamin K_1_ and K_2_ was below the values reported in healthy European reference populations and intake of both forms was particularly low on HD treatment days [6]. While the exact reason for the low intake is unknown, it is likely related to the dietary regimen prescribed for HD patients and overall poor nutrient intake. In addition, plasma levels of dp-ucMGP were elevated above the range reported in healthy people in all patients and 82.5% had elevated PIVKA-II [6]. However, the levels of these functional biomarkers of vitamin K status did not correlate with the measured dietary vitamin K intake in this small group of patients. Older studies performed in maintenance HD patients suggest that genetic variation (e.g., apolipoprotein E genotype) may be one important contributor [16].

In the Cranenburg study, and in one other large study of maintenance HD patients, levels of dp-ucMGP correlated with PIVKA-II levels suggesting a global vitamin K deficiency in HD patients in the arterial wall and liver respectively [6,17]. We have reported that 29% and 93% of maintenance HD patients met criteria for sub-clinical vitamin K deficiency based on low levels of phylloquinone and high levels of ucOC respectively [3] suggesting there is also vitamin K deficiency at the level of bone. Taken together, there is a growing and consistent body of evidence supporting the notion that maintenance HD patients have a global poor vitamin K status.

One randomized controlled trial has evaluated the response of biomarkers of vitamin K status (dp-ucMGP, PIVKA-II and ucOC) to 3 doses of vitamin K_2_ (MK-7) administered over a period of 6 weeks [5]. This study confirmed that most HD patients have functional vitamin K deficiency at baseline and reported that vitamin K_2_ (MK-7) supplementation decreased dp-ucMGP and PIVKA-II levels. However, only at the highest dose did MK-7 impact upon the carboxylation status of OC. This study provides the first evidence that functional vitamin K deficiency can be treated with vitamin K supplementation in the maintenance HD population, although the doses of each form of vitamin K required to correct the problem are not established.

### 3.3. Vitamin K Status in Patients with CKD Receiving Peritoneal Dialysis (PD)

Dietary vitamin K intake in patients receiving PD has not been assessed. There have been two studies demonstrating that PD patients have a similar degree of vitamin K insufficiency as maintenance HD patients. In a recent study of 28 PD patients, 46% had vitamin K deficiency as measured by elevated PIVKA II levels (>2 nM/L) [18]. We demonstrated in a cross-sectional study of 21 PD patients that 24% of patients had vitamin K deficiency as assessed by serum K_1_ (<0.4 nM/L) and all patients had vitamin K deficiency as assessed by increased percentage of ucOC (>20% was the cut off for vitamin K deficiency, mean was 60%) [19]. PD is an independent and home-based kidney replacement therapy and therefore PD patients tend to be younger and have less co-morbidity then maintenance HD patients. However, maintaining adequate nutrition represents a significant limitation of PD therapy and it is possible that this group of patients is at equivalent, or possibly higher, risk among the CKD population.

### 3.4. Studies of Bone Metabolism and Fracture Risk

Osteocalcin is a vitamin K dependent protein secreted by osteoblasts and plays a key role in maintaining appropriate bone turnover. Conditions associated with deficiency of vitamin K may lead to an increase in bone fracture risk and treatment with vitamin K may prevent bone loss and reduce fracture risk [20]. Patients with CKD requiring dialysis have a 4-fold increased risk of hip fracture compared with the general population. The Dialysis Outcomes and Practice Patterns study conducted in 12 countries reported an incidence of 8.9 new hip fractures and 25.6 new fractures of any kind per 1000 patient-years among maintenance HD patients [21]. Kohlmeier *et al.*, were the first to demonstrate an independent association between poor vitamin K status and risk of bone fracture in patients who had reached ESKD and who required hemodialysis [4]. More recently, Fusaro *et al.*, conducted an observational study of 387 prevalent HD patients and, using spinal radiographs, determined that over 50% of prevalent hemodialysis patients have vertebral fractures [22]. In this study, vitamin K_1_ deficiency (defined as a phylloquinone level adjusted for serum triglycerides <0.21 ng/mL), was the strongest predictor for the presence of a vertebral fracture with an adjusted odds ratio approaching 3 [22]. A few small studies have evaluated the impact of vitamin K supplementation on biomarkers of bone status in CKD patients. Only one study has considered vitamin K_1_ supplementation. Phylloquinone (2 mg/day) did not change levels of parathyroid hormone (PTH) or bone-specific alkaline phosphatase (ALP) over 6 months of treatment in a small group of maintenance HD patients [23]. The impact of phylloquinone on the carboxylation status of OC has not been reported. Two studies evaluated the impact of vitamin K_2_. In a German study, 53 maintenance HD patients were randomized to 3 dosages of vitamin K_2_ (MK-7; 45 µg/day, 135 µg/day, and 360 µg/day) treatment for a period of 6 weeks [5]. Only the highest dosage of MK-7 (360 µg/day) resulted in a significant decrease in ucOC. The impact of MK-7 supplementation on other biomarkers of bone metabolism was not reported in this study. Sugimoto reported that vitamin K_2_ (MK-4; 45 mg/day) resulted in a reduction of bone-specific alkaline phosphatise (ALP) over a 12 month period in 8 stable PD patients [24]. Taken together, a high dose of vitamin K_2_ was found to have modest impact upon the level of circulating ucOC and other bone-related markers, but whether this translates to changes in bone density or a reduction in fracture risk in the CKD population was not studied.

By inhibiting the recycling of vitamin K, warfarin induces a vitamin K deficient state which results in the inhibition of vitamin K-dependent proteins. Fusaro *et al.*, found that hemodialysis patients treated with warfarin for greater than 1 year had an increased risk of vertebral fractures compared to those not on warfarin [25].

### 3.5. Studies of Vascular Calcification

#### Observational Studies of CAC

The presence of coronary artery calcification (CAC) is associated with an increased risk of cardiovascular disease and mortality independent of other risk factors [26,27,28]. Patients with CKD are at markedly increased risk for CAC [29,30,31]. MGP, a key inhibitor of vascular calcification, is present in the arterial wall and is dependent on vitamin K for its activity. Insufficient vitamin K availability may lead to an increase in under-carboxylated, and therefore inactive, MGP. The risk of vascular calcification in individuals is presumed higher if the calcification-inhibitory effect of MGP is impaired. 

Pre-clinical studies confirm an influence of a diminished vitamin K status (e.g., warfarin or low dietary K_1_) on the severity of arterial calcification in rats with CKD that can be prevented by a diet enhanced with vitamin K_1_ [9]. However, the impact of vitamin K status and the carboxylation status of MGP specifically, on calcification in patients with CKD has not been resolved. It has been found that total ucMGP levels are significantly lower in patients on dialysis compared to control subjects [22,32]. However, in addition to carboxylation, MGP must also be phosphorylated on three serine residues to be secreted and fully active [33]. Therefore, to address this issue, some studies have measured the de-phosphorylated ucMGP (dp-ucMPG) form and have found that it is increased in CKD [11] and dialysis patients [6]. Preliminary studies in humans with CKD demonstrated that dp-ucMGP levels rise progressively with the severity of CKD [11]. Further, the dp-ucMGP levels were independently associated with aortic calcification measured by abdominal CT as well as mortality over a median duration of follow-up of 846 days [11,32]. These studies support an influence of vitamin K status on calcification in this population. However, whether the carboxylation status of MGP in the circulation represents a surrogate marker for vascular calcification in CKD (*i.e*., higher ucMGP:cMGP ratio) cannot be determined from these studies. Westenfeld *et al.*, have reported that levels of dp-ucMGP are sensitive to vitamin K_2_ (e.g., MK-7) supplementation in maintenance HD patients [5]. Whether or not improved vitamin K status translates into a reduction in vascular calcification is still unknown.

Warfarin is a vitamin K antagonist and its use represents a model of vitamin K insufficiency. We have demonstrated that long-term treatment with a vitamin K antagonist, warfarin, is independently associated with greater severity of aortic valve calcification in maintenance HD patients [34]. In addition, warfarin has been anecdotally linked to calciphylaxis in HD patients. Despite these associations, and the established increased risk of bleeding linked to this drug in the setting of CKD, warfarin is still prescribed to approximately 20% of dialysis patients in North America [35].

## 4. Clinical Trials

There is randomized controlled evidence from the general population that supports a treatment effect of vitamin K on arterial calcification. Supplementation with daily phylloquinone (0.5 mg) slowed the progression of CAC over 3 years in healthy older adults with pre-existing CAC at baseline [36]. Similarly, in an earlier RCT, post-menopausal women who were randomized to receive a supplement containing 1 mg/day phylloquinone in addition to minerals and 320 IU vitamin D_3_ had better carotid artery distensibility, compliance, and elasticity after three years, compared to women who received the mineral supplement alone or the mineral supplement with vitamin D_3_ [37]. Both of these trials studied individuals who were not at any particular risk for sub-clinical vitamin K deficiency. 

It is well established that patients with kidney failure have an increased risk for both vascular calcification and sub-clinical vitamin K deficiency; yet, to date, no trial has been completed that examines whether vitamin K supplementation prevents the progression of calcification in this population. However, two randomized, placebo-controlled trials in this patient population are currently in progress and registered with Clinicaltrials.gov. The European study (VitaVasK study) is randomizing prevalent hemodialysis patients to 5 mg of vitamin K_1_ thrice weekly for 18 months. The Canadian study (iPACK-HD study) is randomizing incident hemodialysis patients (<6 months) to 10 mg of vitamin K_1_ thrice weekly for 12 months.

## 5. Conclusions

It has been consistently demonstrated that patients with CKD as well as those on HD or PD have subclinical vitamin K deficiency, as indicated by reduced serum K_1_, higher percentage of ucOC and higher dp-ucMGP, as well as increased levels of PIVKA-II. Therefore this is a patient group at risk for the many consequences of sub-clinical vitamin K deficiency including cardiovascular disease and bone fracture, and vitamin K status may be a modifiable risk factor for these outcomes. Biomarkers of vitamin K status appear sensitive to supplementation in this population. These data, and the results of two clinical trials in progress, may inform clinical practice in the future.
